# Interference Competition for Mutualism between Ant Species Mediates Ant-Mealybug Associations

**DOI:** 10.3390/insects11020091

**Published:** 2020-02-01

**Authors:** Yongheng Liu, Chong Xu, Qiuling Li, Aiming Zhou

**Affiliations:** Hubei Insect Resources Utilization and Sustainable Pest Management Key Laboratory, College of Plant Science and Technology, Huazhong Agricultural University, Wuhan 430070, China; yonghengliu@webmail.hzau.edu.cn (Y.L.); hzauxuchong@163.com (C.X.); 17537161706@163.com (Q.L.)

**Keywords:** ant-mealybug mutualism, ant interference competition, *Phenacoccus solenopsis*, natural enemies

## Abstract

Ant-hemipteran mutualism has been well documented, and many studies have reported the interference competition between ant species for the mutualism. However, little is known on how this interference competition impacts the reciprocally beneficial association. Previous studies demonstrated that the invasive mealybug *Phenacoccus solenopsis* (Tinsley) has established close mutual relationship with the ghost ant *Tapinoma melanocephalum* (Fabricius). The sympatric ants, *Paratrechina longicornis* (Latreille) and *Tetramorium bicarinatum* (Nylander) were frequently observed to compete for nutrient honeydew produced by *P. solenopsis* with *T. melanocephalum*. Herein, we investigated the effects of interference competition between the ant species on the ant-mealybug interactions. *Phenacoccus solenopsis* benefited from the tending by *T. melanocephalum* and *P. longicornis*. Interference competition between *T. melanocephalum* and *P. longicornis* interrupted the mutualism, suppressed the trailing activity of both species, but negligibly influenced the parasitism of *Aenasius bambawalei* Hayat, a solitary endoparasitoid of *P. solenopsis*. *Harmonia axyridis*, a predator of *P. solenopsis*, showed a significant avoidance when encountering with *T. melanocephalum* or *P. longicornis*, but not *T. bicarinatum*. Ant workers showed higher aggressiveness and lower exploratory activity when *T. melanocephalum* encountered *P. longicornis*. However, competition between *T. melanocephalum* and *T. bicarinatum* seldom influenced the trailing and exploratory activity of *T. melanocephalum*. It is concluded that interference competition for mutualism between ant species can mediate ant-mealybug associations and the fitness of mealybug colony. Our results also demonstrate that the effects of interference competition between ant species on ant-mealybug mutualism are varied among ant species.

## 1. Introduction

Mutualism, which is defined as the reciprocally beneficial interaction between two species, is an important ecological interaction [1,2]. Mutualism between ants and hemipterans is common in natural environment and plays key roles in ecological processes [2,3,4]. Ants tend and protect honeydew-producing hemipterans from predators and parasitoids in a wide range of ecosystems [5]. In return, hemipterans provide ants with honeydew as a crucial food source [6,7]. Currently, the broad ecological effects of ant-hemipteran interactions have been increasingly recognized and documented [7,8,9,10,11]. The mutual relationship would be stable when the trade-off between the cost and benefit is in balance [12]. However, this mutual relationship can be strongly influenced by the local biotic neighborhoods in which it occurs [13]. The beneficial effects of mutualist species on each other can vary along with the changes in biotic factors, such as the density of associated species, seasonality, spatial distribution, identity of species involved and quality of host plants [13,14,15,16,17,18]. The outcome of the interactions can vary from mutualistic to commensalistic or even antagonistic, depending on the ecological context and the interacting species [19].

Although many studies have elucidated the ant-hemipteran interactions and their ecological outcomes, relatively few studies have explored the various outcomes resulting from the competitive mutualism among the interactional species. It is accepted that competition is an important force in natural communities [20,21]. Previous studies have reported the competition among hemipterans to exploit the same resources for the mutualistic services of ants [22,23]. Protection efficiency of *Lasius niger* (Linnaeus) differs among aphid species [24]. Ants usually preferentially tend the aphid species that produce more honeydew or excrete ant-preferred sugars [21,25]. Subsequent studies have revealed that the recruitment response of ants is directly influenced by the quality and quantity of honeydew [26,27,28]. These results suggest that the effect of the competition may depend on the relative attractiveness of aphids to ant species involved in the mutual interactions. 

In comparison with the competition for ant tending among different hemipterans, the interference competition between ant species for mutualist-provided resources has been less studied. Such competition is particularly acute between invasive and native ant species. Intense interspecific competition between *Solenopsis invicta* Buren and native ants for carbohydrate sources from honeydew-producing hemipterans widely exists in both its native and invasion regions [4]. *Solenopsis invicta* suppresses native ants by monopolization and competition for mutualism with honeydew-producing hemipterans [29,30,31]. Besides, interspecific competition from ecologically dominant native ants can affect the invasion success of invasive species [4]. Due to its advantages in competition for space and food exploitation, native ant *Tapinoma nigerrimum* (Nylander) notably reduces the likelihood of incipient colony establishment and survival of Argentine ant *Linepithema humile* Mayr [32]. Honeydew-producing citrus mealybug, *Pseudococcus citriculus* Green, can associate with various mutualistic ant species, including *Lasius niger* and *Pristomyrmex pungens* Mayr. The territories of these two ant species are completely separated, and there are occasional territory takeovers of one ant species by the other. *Lasius niger* is considered as a more effective mutualistic partner than *P. pungens* because of its higher aggressiveness [33]. These results suggest that interference competition between ant species may be an important index to evaluate the intensity of ant-hemipteran association. Previous studies have described the interference competition between ant species for mutualist-provided carbohydrates [7,29,34]. However, it remains unclear how the interference competition affects involved ant communities, including foraging activity and aggression. More importantly, the direct effects of the interference competition on outcome of the mutual association are largely unknown.

The mealybug *Phenacoccus solenopsis* Tinsley (Hemiptera: Pseudococcidae) is a polyphagous invasive species in China. It has established widely mutualistic relationships with a wide range of ant species, including *S. invicta*, *T. melanocephalum*, *P. longicornis*, *T. bicarinatum* and *Pristomyrmex pungens* in ecosystems [35,36,37,38]. The ghost ant *T. melanocephalum* is one of the most dominant native ant species in the areas invaded by *S. invicta* [39]. *Tapinoma melanocephalum* also competes for honeydew produced by mealybugs with other native ant species [31]. Currently, few studies have reported the effects of the interference competition between the native ant species, including *T. melanocephalum*, *P. longicornis* and *T. bicarinatum*, on ant-mealybug interactions. Understanding the response of each partner in the beneficial associations to ant competition is critical to clarifying how resource competition between ant species regulates the ecological interactions. Herein, we conducted a series of laboratory experiments to test the effect of ant interference competition on the colony growth of *P. solenopsis* and the performance of natural enemies of mealybugs. We also evaluated how interference competition mediates the trailing activity, aggressiveness index and exploratory behavior of these three ant species. We hypothesize that *T. melanocephalum* competes with *P. longicornis* and *T. bicarinatum* for mutualist that provides carbohydrate resources. The interference competition between ant species would affect the outcome of ant-mealybug mutualism. Our results may provide more accurate information for better understanding the mechanism by which interspecific competition among ant species mediates ant-hemipteran mutualism. 

## 2. Materials and Methods

### 2.1. Plants and Insects

Cotton plants (Jimian 11, non-transgenic, Academy of Agriculture and Forestry Sciences, Hebei, China) were grown in soil media (organic matter ≥20%; Jiangsu Peilei Technology Development CO., LTD) within plastic flowerpots. Each plant used in the experiment was approximately 30–40 cm in height with 15–20 true leaves. *Phenacoccus solenopsis* colonies were collected from a cotton field in Huazhong Agricultural University and transferred to cotton plants in flowerpots. Sixty 1st instar mealybug nymphs were placed on cotton plants and raised for several generations in the laboratory at the temperature of 26 ± 2 °C and relative humidity of 60–70%. The parasitoid *Aenasius bambawalei* and predator *Harmonia axyridis* were also collected from cotton field. Both parasitoid and predator colonies were fed mealybug nymphs in the laboratory at 26 ± 2 °C and under an LD 16:8 h photoperiod. 

Sympatric ant colonies of *Tapinoma melanocephalum*, *Paratrechina longicornis* and *Tetramorium bicarinatum* were collected from the suburb of Wuhan. Ant colonies were separated from the soil by dripping water into the plastic boxes until the colonies floated [40]. Ants were then removed and reared in plastic boxes with tubes filled with distilled water. The colonies were subsequently divided into several small colony fragments by weight using a microbalance (Sartorius BSA 224S, Elk Grove, IL, USA). Ant colony fragment of each species consisted of 1 queen and approximately 2500 workers. Each ant colony fragment was placed in a 9-cm plastic Petri dish and then introduced into a plastic container (22 × 15 × 7 cm) as an artificial nest [17]. Each ant colony fragment was only used once in all experiments. Ant colonies were supplied weekly with live mealworm larvae (*Tenebrio molitor* L.; Coleoptera: Tenebrionidae) and 50 mL 10% honey water solution. All ant colonies were reared in the laboratory at 26 ± 2 °C and relative humidity of 60–70%.

### 2.2. Effects of Ant Competition on Mealybug Colony Growth under Predation

Each potted cotton plant was held in a plastic tray (42 × 26 × 18 cm) and covered with nylon netting surrounded by a wooden cage (90 × 90 × 100 cm). The potted plants were randomly arranged. Thirty 2nd instar mealybugs were transferred to each caged plant. The ant colony fragment was placed into a small plastic container (22 × 15 × 7 cm), and connected with the plant tray by a silicone tube (0.8 cm in diameter and 10 cm in length) (Figure S1). To prevent the ants from escape, Teflon (Sigma Aldrich, Shanghai, China) was applied halfway up the inner surface of each ant colony container and plant tray. This treatment design allowed ant workers to approach the mealybug colonies directly through the plant trunk. After 24 h, two lady beetle larvae were introduced on the plants as predators. The ant colony was provided with 50 mL of water every 2 days. A completely randomized design was conducted for this experiment, including *T. melanocephalum*, *P. longicornis*, *T. bicarinatum*, a combination of *T. melanocephalum* and *P. longicornis* colonies, and a combination of *T. melanocephalum* and *T. bicarinatum* colonies. No ant tending was used as a control. Each treatment was replicated 12 times, and the number of live mealybugs per plant was recorded after six weeks. The colony growth rate of mealybugs was defined as the final population density divided by the initial population density. 

### 2.3. Effects of Ant Competition on Parasitism of Mealybugs by A. bambawalei

The effects of ant competition on the parasitism of *A. bambawalei* were also evaluated by the caged bioassay described above. We transferred the 3rd instar mealybugs to caged plants (60 individuals per plant). The ant colony fragment was then connected with the plant tray. The ants were given two mealworms and 50 mL water every two days. Two fertilized female parasitoids were released on each plant after 24 h. Five treatments of ant-tending and a control were implemented as described in 2.2. Each treatment was replicated 12 times. All surviving mealybugs and mummified mealybugs on each plant were collected and counted after two weeks. The parasitism percentage was defined as the number of mummified mealybugs divided by the total number of mealybugs on each plant. 

### 2.4. Effects of Interference Competition on Ant Trailing Activity

The trailing activity of each ant species was determined in laboratory assay. One cotton plant was placed in the plastic tray. The 3rd instar mealybugs were introduced to the cotton plants (60 individuals per plant) and allowed to acclimate and feed for 24 h. The ant colony fragment was deprived of any carbohydrate source for 12 h and then connected with the plant tray. Workers could enter into the plant tray and move toward the plant through the outside of the pot. Ant tending treatments were conducted as follows: *T. melanocephalum*; *P. longicornis*; and a combination colony of *T. melanocephalum* and *P. longicornis*. The similar experiments were repeated with *T. melanocephalum*; *T. bicarinatum*; and a combination colony of *T. melanocephalum* and *T. bicarinatum*. Each treatment was replicated 15 times. The trailing activity of ants was evaluated on the plants by counting the number of trailing ants moving up and down the plant trunk for 5 min after 24 h. 

### 2.5. Effects of Interference Competition on Predator Performance

The effects of ant competition on the performance of lady beetles were tested by using a behavior observation apparatus described previously [38,41]. Lady beetle females were provided choices to forage and lay eggs in two arenas, in one of two jointed plastic boxes (40 × 25 × 30 cm). One arena was occupied by ants, and ants were excluded from the other arena. Each box has an open window in the upper half of the jointed wall. Teflon was applied halfway up the inside of each box below the window to prevent ants from crossing from one area into the other. Lady beetle adults could still have access to both arenas by climbing or flying across the open window (Figure S2). A 14-cm Petri dish coated with Teflon was placed in the center of each box, and a cotton leaf with 60 3rd instar mealybugs was placed in the dish. The petiole of a cotton leaf was wrapped with moist cotton to maintain turgor. At the beginning of the experiment, 100 ant workers were placed into each box together with 10 lady beetle adults. Teflon was used to prevent mealybugs from escaping and ants from invading. Five treatments were implemented to determine the effects interference competition on predator performance: *T. melanocephalum*/no ants; *P. longicornis*/no ants; *T. bicarinatum*/no ants; *T. melanocephalum*-*P. longicornis*/no ants; and *T. melanocephalum*-*T. bicarinatum* /no ants. Each comparison was replicated 12 times. The number of ladybeetles in each arena and the number of eggs laid in the Petri dishes were counted at after 48 h.

### 2.6. Aggressiveness Evaluation between the Ant Species at Individual Level

The aggressiveness between different ant species at individual level was quantified with the protocol described by Errard and Hefetz [42] and Tsutsui et al. [43]. One ant worker was randomly selected from the three ant species. Two individual workers were paired together in a Petri dish (diameter = 3.5 cm). To prevent the ants from climbing out, the Petri dish was coated with Teflon. All tested individuals were derived from different colonies. The assigned ant workers were allowed to acclimate for one minute in the Petri dish before the test. The behavioral interactions between the workers were scored in 5 min: 1: avoidance (keep away from each other); 2: brief touch (≤1 s); 3: long touch (>1 s); and 4: attack postures (bite). Aggressiveness was determined in both *T. melanocephalum*-*P. longicornis* interaction and *T. melanocephalum*-*T. bicarinatum* interaction. Fifty trials were implemented for each treatment. The aggressiveness index was calculated by the following formula as previously described by Errard and Hefetz [42] (1). The percentage of aggressiveness level was calculated and analyzed by Formula (2):

(1) $$\frac{\sum_{i=1}^{n}\delta_{i}t_{i}}{T}$$

(2) $$\frac{\delta_{i}t_{i}}{\sum_{i=1}^{n}\delta_{i}t_{i}}$$

where *δ_i_* represents the score we evaluated in the attack interaction, *t_i_* means the duration of each behavioral interaction, *n* means the number of replications in each treatment and *T* is the total time for each trial.

### 2.7. Effects of Interference Competition on the Exploratory Activity of Ants 

The exploratory activity of ant species was assessed using a modified apparatus originally described by Grangier and Lester [44]. Three cotton plants were placed in a plastic tray (75 × 44 × 12 cm) and each plant was 20 cm apart. Each plant was considered as a different foraging arena for ant colonies: original arena, medium arena and extended arena (Figure S3). Each cotton plant was infected with 60 3rd instar mealybugs before the test. The mealybugs were allowed to acclimate for 24 h on the plants. The ant colony fragment was then connected with the plant tray. To prevent ant escape, the inside of the tray was treated by Teflon. The outside of the pot located in medium arena and extended arena was also treated by Teflon respectively to discourage foraging path of ant to the plants. This treatment makes the plant located in original arena become the only access for workers to foraging. Each two contiguous plants were connected by a wooden bridge (25 cm length). The exploratory activity of ants on plant in different foraging arenas was scored: 1: original arena; 2: medium arena; 3: extended arena. Exploratory activity of ants in the original arena was defined as the number of workers moving up and down the plant trunk in 5 min. The exploratory activity of the ants in medium arena and extended arena was defined as the number of workers moving toward and away from the plant on bridge 1 and bridge 2, respectively, in 5 min (Figure S3). The exploratory activity was calculated by using the modified formula proposed by Errard and Hefetz [42] (3). The percentage of exploratory level of each ant species was calculated by using formula (4). The treatments were the same as those for trailing activity test described previously in experiment 2.4. Each treatment was repeated by 10 trials. 

*δ_i_* represents the score evaluated in the exploratory behavior; *n_i_* means the number of workers in each foraging arena; *T* is the total time for each trial; and m means the number of replications for each treatment.

(3) $$\frac{\sum_{i=1}^{m}\delta_{i}n_{i}}{T}$$

(4) $$\frac{\delta_{i}n_{i}}{\sum_{i=1}^{m}\delta_{i}n_{i}}$$

### 2.8. Statistical Analysis

For experiment 2.2 and 2.3, to satisfy the precondition of variance analysis, colony growth rate of mealybug was treated by log10-transformation. Parasitism by *A. bambawalei* was treated by the arcsine square root-transformation. One-way ANOVA was used to compare the variance in means of mealybug colony growth rate and parasitism among different ant tending treatments. An *LSD*-test was performed for multiple comparisons of mealybug colony growth rate and parasitism between the ant treatments. For experiment 2.4, an *Independent sample t*-test was used to analyze the difference in ant trailing activity between single colony and combined colony. For experiment 2.5, a *Paired sample t*-test was performed to analyze the difference in number of lady beetle adults and eggs between ant included arena and ant excluded arena. For experiment 2.6, an *Independent sample t*-test was used to determine ant aggressiveness index and aggressiveness level between *T. melanocephalum*-*P. longicornis* competition and *T. melanocephalum*-*T. bicarinatum* competition. For experiment 2.7, an *Independent sample t*-test was used to analyze the difference in ant exploratory activity and exploratory level between single colony treatment and combined colony treatment. All statistical analyses were conducted with SPSS, version 19.0 (SPSS Inc., Chicago, IL, USA).

## 3. Results

### 3.1. Mealybug Colony Growth under Predation

**Figure 1:**
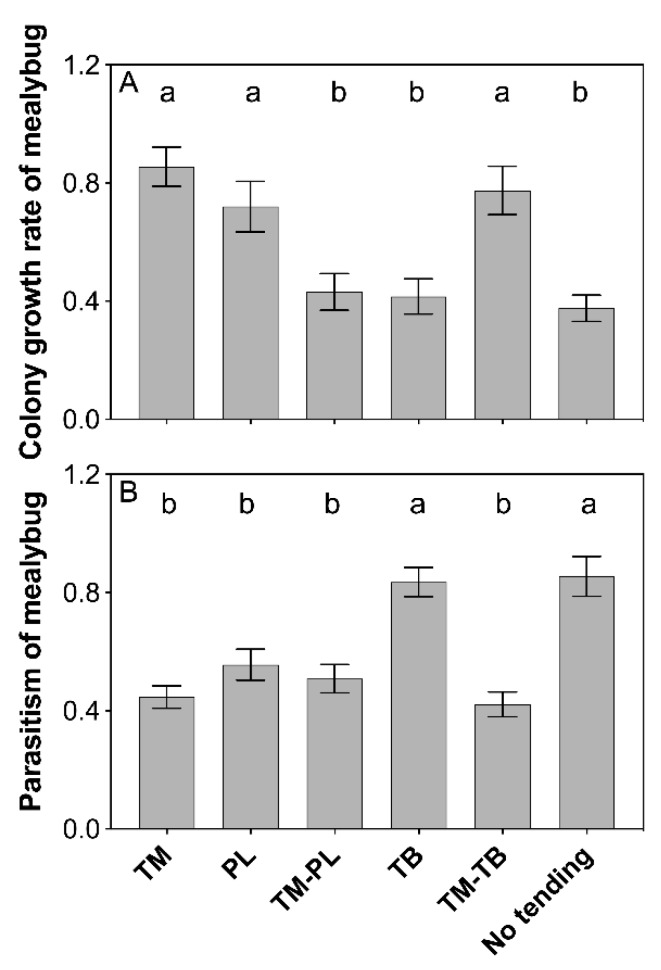
Effects of interference competition on mealybug populations. (**A**) colony growth rate of mealybug; (**B**) parasitism of mealybug. The data are presented as the mean ± SE. Bars sharing the same letters indicate no significant differences among the treatments (*P* > 0.05). Colony growth rate was treated by log10-transformation, and the percent of parasitism on the mealybugs was treated by the arcsine square root-transformation. *Tapinoma melanocephalum*, *Paratrechina longicornis*, *Tetramorium bicarinatum*, *Tapinoma melanocephalum*-*Paratrechina longicornis* confrontation and *Tapinoma melanocephalum*- *Tetramorium bicarinatum* confrontation were abbreviated into TM, PL, TB, TM-PL and TM-TB, respectively.

The results showed that the growth of mealybug colony was significantly different under the tending by different ant species in the presence of predation (F_5, 66_ = 9.603, P < 0.001). Compared with no ant tending, mealybug colony growth was significantly improved by tending of *T. melanocephalum*, *P. longicornis* and a combination of *T. melanocephalum*-*T. bicarinatum* (Figure 1A, TM: P < 0.001; PL: *P* = 0.001; TM-TB: P < 0.001; *LSD*-test). No positive effects on mealybug colony growth were observed for tending by combination of *T. melanocephalum*-*P. longicornis* or *T. bicarinatum* alone (Figure 1A; TM-PL, P = 0.566; TB, P = 0.680; *LSD*-test). 

### 3.2. Parasitism by the Parasitoids

Percentage of parasitism varied among ant tending treatments (Figure 1B; F_5, 66_ = 14.635, P < 0.001). Compared with no ant tending, parasitism was largely reduced when mealybugs were tended by *T. melanocephalum*, *P. longicornis* and a combination of *T. melanocephalum*-*P. longicornis*, and a combination of *T. melanocephalum*-*T. bicarinatum* (*P* < 0.001, respectively; *LSD*-test), while tending by *T. bicarinatum,* had no significant effect on parasitism (*P* = 0.784; *LSD*-test). 

### 3.3. Ant Trailing Activity

**Figure 2:**
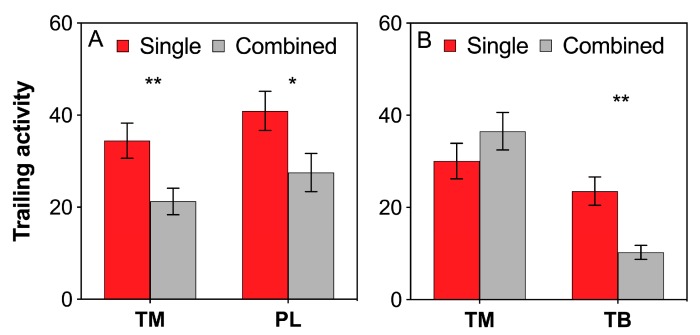
Effects of interference competition on ant trailing activity. (**A**) *T. melanocephalum*-*P. longicornis* interaction; (**B**) *T. melanocephalum*-*T. bicarinatum* interaction. The data are presented as the mean ± SE. Asterisk (* or **) on bars indicates significant differences between the treatments (*P* < 0.05 or *P* < 0.01). *Tapinoma melanocephalum*, *Paratrechina longicornis* and *Tetramorium bicarinatum* were abbreviated into TM, PL and TB, respectively.

Both *T. melanocephalum* and *P. longicornis* exhibited higher trailing activities under single colony treatment than under combined colony treatment (Figure 2A; TM: *t* = 2.900, *df* = 28, *P* = 0.007; PL: *t* = 2.298, *df* = 28, *P* = 0.029; *independent sample t*-test). Furthermore, the trailing activity of *T. melanocephalum* was almost not influenced by interference competition with *T. bicarinatum* (Figure 2B; TM: *t* = −1.319, *df* = 28, *P* = 0.198; *independent sample t-test*); however, that of *T. bicarinatum* rapidly decreased when they encountered *T. melanocephalum* colony (Figure 2B; TB: *t* = 4.507, *df* = 28, *P* < 0.001; *independent sample t*-test). 

### 3.4. *Predator Performance*

**Figure 3:**
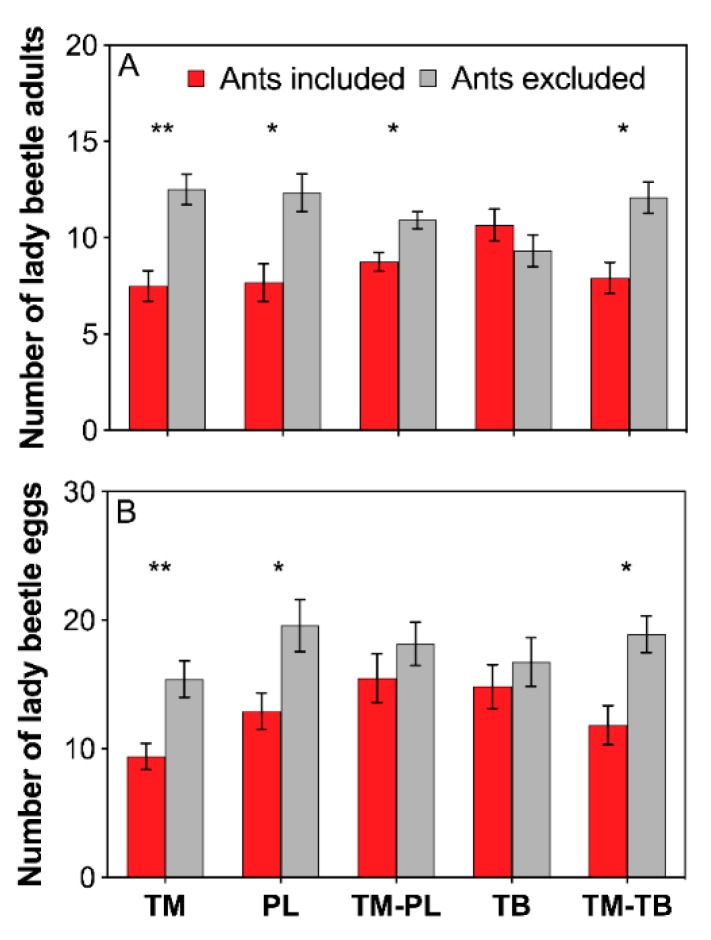
Effects of interference competition on predator performance. (**A**) number of lady beetle adults; (**B**) number of lady beetle eggs. The data are presented as the mean ± SE. Asterisk (* or **) on bars indicates significant differences between ant treatments (*P* < 0.05 or *P* < 0.01). *Tapinoma melanocephalum*, *Paratrechina longicornis*, *Tetramorium bicarinatum*, *Tapinoma melanocephalum*-*Paratrechina longicornis* confrontation and *Tapinoma melanocephalum*- *Tetramorium bicarinatum* confrontation were abbreviated into TM, PL, TB, TM-PL and TM-TB, respectively.

Lady beetle adults apparently avoided the foraging arena occupied by workers of *T. melanocephalum*, *P. longicornis*, *T. melanocephalum*-*P. longicornis*,and *T. melanocephalum*-*T. bicarinatum* combined colonies (Figure 3A; TM: *t* = −3.153, *df* = 11, *P* = 0.009; PL: *t* = −2.401, *df* = 11, *P* = 0.035; TM-PL: *t* = −2.493, *df* = 11, *P* = 0.030; TM-TB: *t* = −2.597, *df* = 11, *P* = 0.025; *paired sample t*-test). Tending by *T. bicarinatum* had little effect on the predation of lady beetles (Figure 3A; TB: *t* = 0.813, *df* = 11, *P* = 0.433; *paired sample t*-test). Lady beetles produced fewer eggs in the arenas with workers of *T. melanocephalum*, *P. longicornis* and a combination of *T. melanocephalum*-*T. bicarinatum* (Figure 3B; TM: *t* = −3.806, *df* = 11, *P* = 0.003; PL: *t* = −2.332, *df* = 11, *P* = 0.040; TM-TB: *t* = −2.858, *df* = 11, *P* = 0.016; *paired sample t*-test). Oviposition of lady beetles was not significantly influenced by *T. bicarinatum* or combination of *T. melanocephalum*- *P. longicornis* (Figure 3B; TB: *t* = −0.879, *df* = 11, *P* = 0.398; TM-PL: *t* = −0.617, *df* = 11, *P* = 0.550; *paired sample t*-test). 

### 3.5. *Ant Aggressiveness*

**Figure 4:**
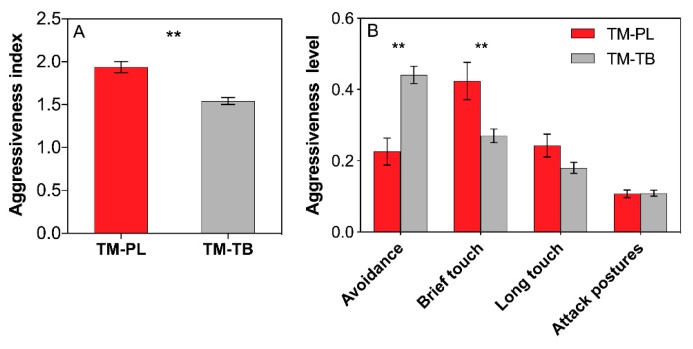
Effects of interference competition on ant aggressiveness. (**A**) aggressiveness index; (**B**) percentage aggressiveness level. The data are presented as the mean ± SE. Asterisk (**) on bars indicate indicates significant differences between the ant treatments (*P* < 0.01). *Tapinoma melanocephalum*-*Paratrechina longicornis* confrontation and *Tapinoma melanocephalum*- *Tetramorium bicarinatum* confrontation were abbreviated into TM-PL and TM-TB, respectively.

The aggressiveness index between *T. melanocephalum* and *P. longicornis* was definitely higher than that between *T. melanocephalum* and *T. bicarinatum* (Figure 4A, *t* = 5.147, *df* = 28, P < 0.001; *independent sample t*-test). Aggressive acts in avoidance occurred more frequently in *T. melanocephalum*-*T. bicarinatum* interaction, while brief touch occurred more frequently in *T. melanocephalum*-*P. longicornis* interaction (Figure 4B; *t* = −4.796, *df* = 28, P < 0.001; *t* = 2.772, *df* = 28, P = 0.01, respectively; *independent sample t*-test). 

### 3.6. Ant Exploratory Activity

**Figure 5:**
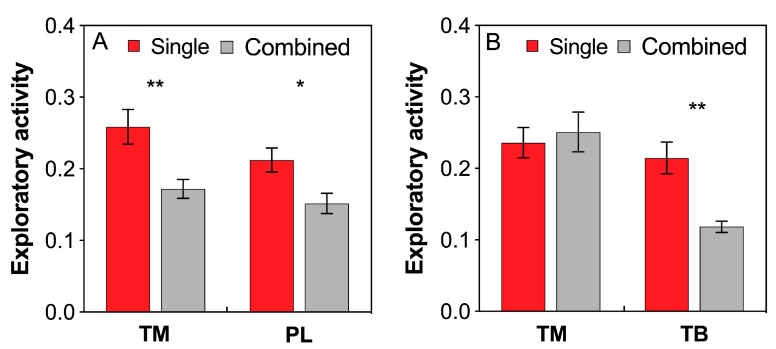
Effects of interference competition on ant exploratory activity. (**A**) *T. melanocephalum*-*P. longicornis* interaction; (**B**) *T. melanocephalum*-*T. bicarinatum* interaction. The data are presented as the mean ± SE. Asterisk (* or **) on bars indicates significant differences between the treatments (*P* < 0.05 or *P* < 0.01). *Tapinoma melanocephalum*, *Paratrechina longicornis* and *Tetramorium bicarinatum* were abbreviated into TM, PL, and TB respectively.

Both *T. melanocephalum* and *P. longicornis* workers showed much higher exploratory activities in single colony than in combined colony treatment (Figure 5A; TM: *t* = 3.153, *df* = 18, P = 0.006; PL: *t* = 2.756, *df* = 18, P = 0.013, respectively; *independent sample t*-test). Interference competition between *T. melanocephalum* and *T. bicarinatum* significantly reduced the exploratory activity of *T. bicarinatum* but not *T. melanocephalum* (Figure 5B; TB: *t* = 4.067, *df* = 18, P = 0.001; TM: *t* = −0.431, *df* = 18, P = 0.672, respectively; *independent sample t*-test). 

**Figure 6:**
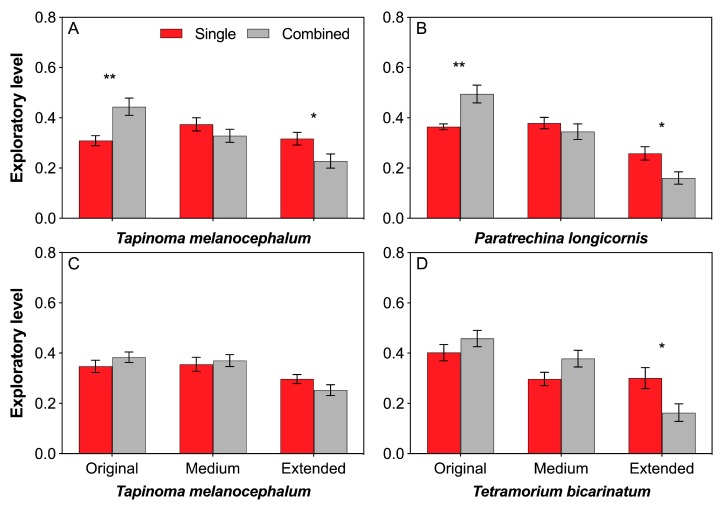
Effects of interference competition on ant exploratory level. (**A**,**B**) *T. melanocephalum*-*P. longicornis* interaction, (**A**) exploratory level of *T. melanocephalum*; (**B**) exploratory level of *P. longicornis*. (**C**,**D**) *T. melanocephalum*- *T. bicarinatum* interaction, (**C**) exploratory level of *T. melanocephalum*; (**D**) exploratory level of *T. bicarinatum.* The data are presented as the mean ± SE. Asterisk (* or **) on bars indicates significant differences between the treatments (*P* < 0.05 or *P* < 0.01).

*T. melanocephalum* possessed higher exploratory level in original arena but lower exploratory level in extended arena when treated by combination of *T. melanocephalum* and *P. longicornis* (Figure 6A; *t* = −3.375, *df* = 18, P = 0.003; *t* = 2.342, *df* = 18, P = 0.031, respectively; *independent sample t*-test). The same case of *P. longicornis* was observed when treated by combined colony (Figure 6B; *t* = −3.559, *df* = 18, P = 0.002; *t* = 2.690, *df* = 18, P = 0.015, respectively; *independent sample t*-test). No significant difference in exploratory level of *T. melanocephalum* was found between single and combination of *T. melanocephalum*- *T. bicarinatum* in each foraging arena (Figure 6C; *t* = −1.135, *df* = 18, P = 0.271; *t* = −0.409, *df* = 18, P = 0.687; *t* = 1.581, *df* = 18, P = 0.131, respectively; *independent sample t*-test). *T. bicarinatum* showed lower exploratory level in extended arena when treated by combination of *T. melanocephalum* and *T. bicarinatum* (Figure 6D; *t* = 2.536, *df* = 18, P = 0.021; *independent sample t*-test). 

## 4. Discussion

Animals usually experience imbalance of food resources in the ecosystem, which often restricts colony abundance and limit the maximum fitness [45]. Honeydew produced by hemipterans is considered as an important carbohydrate resource for many honeydew collection ants [7]. Due to its sugar and amino acid composition, honeydew is essential for ant colony growth and survival [46,47]. Furthermore, the benefits of mutualist-provided carbohydrates to the successful invasion of ants have been extensively studied [4,29,48,49]. Although many studies have illustrated the interference competition between invasive and native ants for the limited carbohydrates produced by hemipterans, few studies have examined the competition between native ant species, and it remains unclear how this interference competition affects the outcome of ant-hemipteran mutualism. This study for the first time provided experimental evidence that the interference competition between native ant species mediates the outcome of beneficial services of ants.

Intensity of ant-hemipteran interactions varies with ant species. When tending the same hemipteran species, different ant species may have different levels of aggressiveness against the enemies of hemipterans and thereby different extents of interference with prey consumption by predators and oviposition by parasitoids [33,50]. *Lasius japonicus* and *P. pungens* differ in aggressiveness towards *Aphis spiraecola* enemies. Compared with *L. japonicas*, *P. pungens* shows lower aggressiveness, and thus, has a lower extent of interference with parasitoid oviposition [51]. Our results showed that the colony growth of *P. solenopsis* was improved by *T. melanocephalum* and *P. longicornis* tending. However, the positive effects of ant tending on mealybug colony growth disappeared when two ant species competed for mutualism, suggesting that the interference competition between two native ant species restrains their mutualistic efficiency. In addition, the tending ant species significantly affected the percentage parasitism by parasitoids, with more mealybug mummies in *T. bicarinatum* tended colonies, which also suggests that *T. melanocephalum* and *P. longicornis* take more aggressive actions against parasitoids than *T. bicarinatum*. Unexpectedly, we found that *T. melanocephalum*-*P. longicornis* and *T. melanocephalum*-*T. bicarinatum* competitions did not influence the parasitism of parasitoids. This result may be attributed to the distinctive approach of *T. melanocephalum* against parasitoids compared with that of other native ants. Workers of *T. melanocephalum* could effectively utilize their pygidial gland secretions as an alarm-defense system during aggressive confrontations [52]. *A. bambawalei* showed strong avoidance to pygidial gland secretions of *T. melanocephalum* [53]. Therefore, repression of parasitism by *A. bambawalei* was also observed even when there was intense competition between *T. melanocephalum* and *P. longicornis* because of the presence of *T. melanocephalum* secretions.

The intensity of interference competition between ant speices can be revealed by the foraging activity of ant workers [29,54]. Honeydew exploitation by *T. melanocephalum* significantly declined when *S. invicta* was introduced, and the foraging activity of *T. melanocephalum* increased when fire ants were excluded [31]. Prevalence of dolichoderine ant *Dorymyrmex bureni* declined in areas invaded by *S. invicta* due to the competition for mutualist-provided carbohydrates [29]. In our study, interference competition reduced the recruitment of *T. melanocephalum*, *P. longicornis* and *T. bicarinatum* for carbohydrates produced by mealybugs. However, the trailing activity of *T. melanocephalum* was not influenced by *T. melanocephalum* and *T. bicarinatum* interaction, suggesting a stronger antagonism action between *T. melanocephalum* and *P. longicornis* than between *T. melanocephalum* and *T. bicarinatum*. This result was also confirmed by subsequent aggressiveness test, which revealed a great passivity of the *T. bicarinatum* workers during confrontation with *T. melanocephalum* workers. Furthermore, the performance of predators was definitely impeded by *T. melanocephalum* and *P. longicornis*, but not by *T. bicarinatum*. Competition between *T. melanocephalum* and *T. bicarinatum* did not obviously affect the negative effects of *T. melanocephalum* on predators, which also suggests that the competitiveness in *T. melanocephalum* and *T. bicarinatum* interaction is relatively low and the protection effect on mealybugs depends on ant species. A similar study reported that due to the more effective patrolling of *L. humile* than *Tapinoma sessile* on plant surfaces to protect honeydew-producing hemipterans, *Aphis gossypii* was less negatively affected by enemies when tended by *L. humile* [55]. Different ant species may have significantly different protection effects on myrmecophilous aphids from natural enemies, which may depend on the potential level of aggressiveness of ants and their foraging strategies when collecting honeydew [56]. Predators produce fewer eggs in arenas occupied by *T. melanocephalum* or *P. longicornis*, but no noticeable difference was observed in arenas with *T. melanocephalum* and *P. longicornis* interaction, suggesting that the negative effects of *T. melanocephalum* or *P. longicornis* on predators were weakened by the interference competition. 

Carbohydrates serve as a principal metabolic fuel, and carbohydrate scarcity may hinder the aggressive activity of ants to a greater extent than deficiency of protein or other nutrients essential for their growth. Invasive ants and co-occurring native ants showed significantly different behavior responses under competition for limited food resource [57]. Carbohydrate scarcity limits the aggression and activity of invasive ant *Linepithema humile* [34]. However, the foraging activity and aggressiveness of the native ant *Prolasius advenus* were increased under carbohydrate scarcity [44]. Our results revealed a significant difference in aggressiveness index between the two pairs of antagonistic competition. The differences in aggressiveness of ants may impact their competitive ability for food resources. Interference competition reduced the exploratory level of the three ant species, particularly that of *T. bicarinatum*. The number of trailing workers of *T. bicarinatum* showed a sharp decline in *T. melanocephalum* and *T. bicarinatum* combined colony compared with a single colony. These results suggest that competition between ant species for honeydew resource produced by hemipterans may inhibit their protection of honeydew producing insects. 

## 5. Conclusions

In conclusion, our results showed different efficiencies of two ant interference competition models on the outcome of mutual interactions. The dominant native ant *T. melanocephalum* competes for access to mutualist-provided carbohydrates with *P. longicornis* and *T. bicarinatum* and suppresses *T. bicarinatum* from this critical resource. Additionally, our results provide novel evidence that competition for mutualist-provided carbohydrates between native ant species can enhance the performance of natural enemies, and in turn affecting the outcome of ant-mealybug mutualism, which may impact on the fitness and invasion process of the invasive mealybug. Currently, most conclusions are based on laboratory evidence, more studies conducted in field are needed to confirm this perspective. These findings may promote our understanding of the significance of interference competition in mediating ecological interactions and arthropod community.

## Supplementary Materials

The following are available online at https://www.mdpi.com/2075-4450/11/2/91/s1.
