# Supplementary material for: Interference Competition for Mutualism between Ant Species Mediates Ant-Mealybug Associations
Source: Insects. 2020 Feb 1;11(2):91. doi: 10.3390/insects11020091 (PMC7073949; doi:10.3390/insects11020091)
Supplement: Supplementary file 1 [file insects-11-00091-s001.pdf]

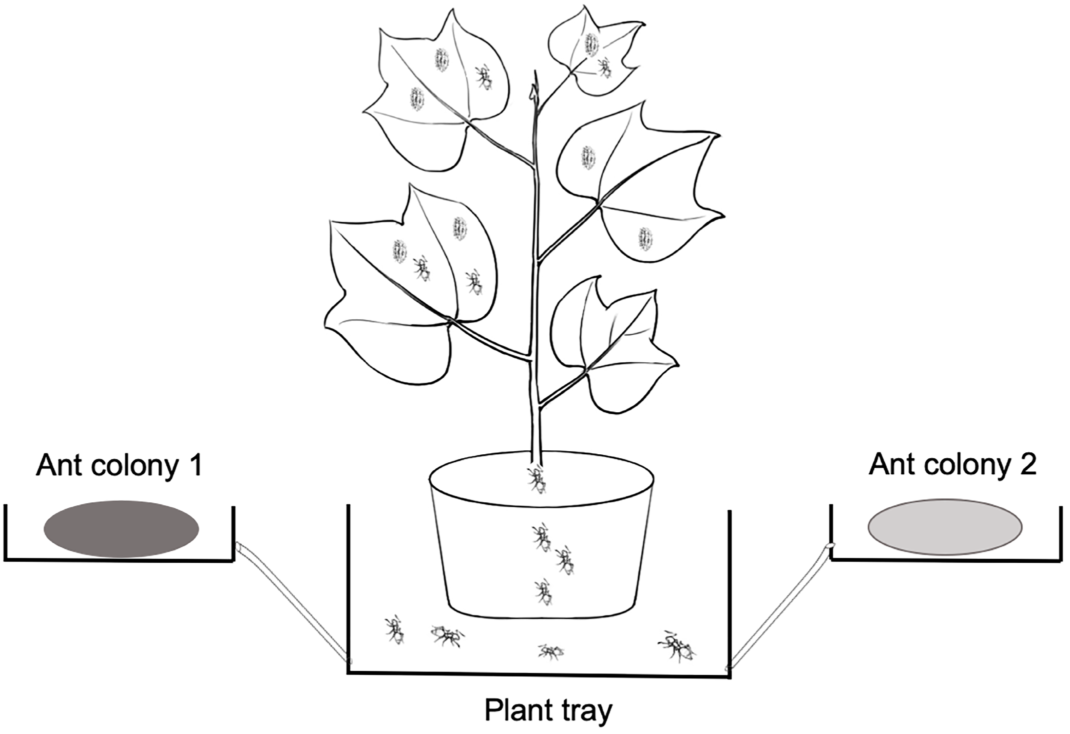

Figure S1 Graphical representation of apparatus used in experiment 2.2. The reconstructed ant colony was connected with the plant tray by a silicone tube, which makes workers could go through the tube and enter into the plant tray. This apparatus was used to test the effects of ant interference competition on colony growth of mealybugs, which was described in experiment 2.2.

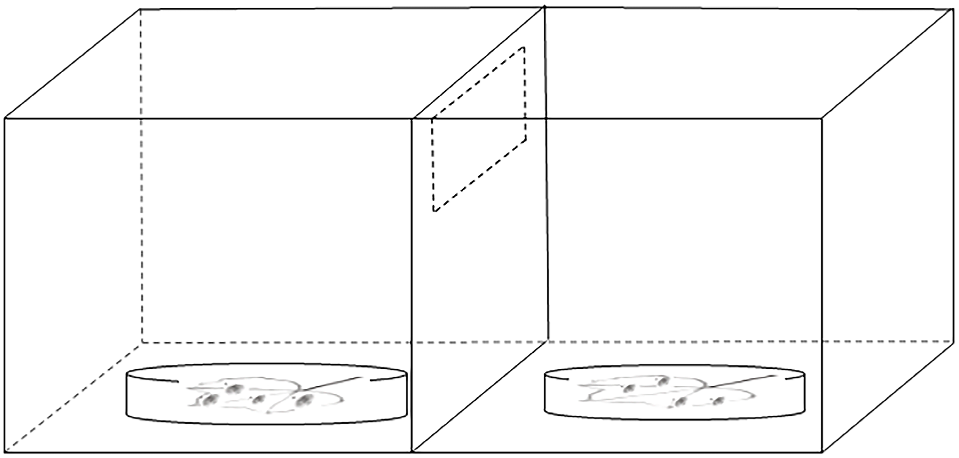

Figure S2 Graphical representation of apparatus used in experiment 2.5. This apparatus was used to test the effects of ant interference competition on predator performance, which was described in experiment 2.5.

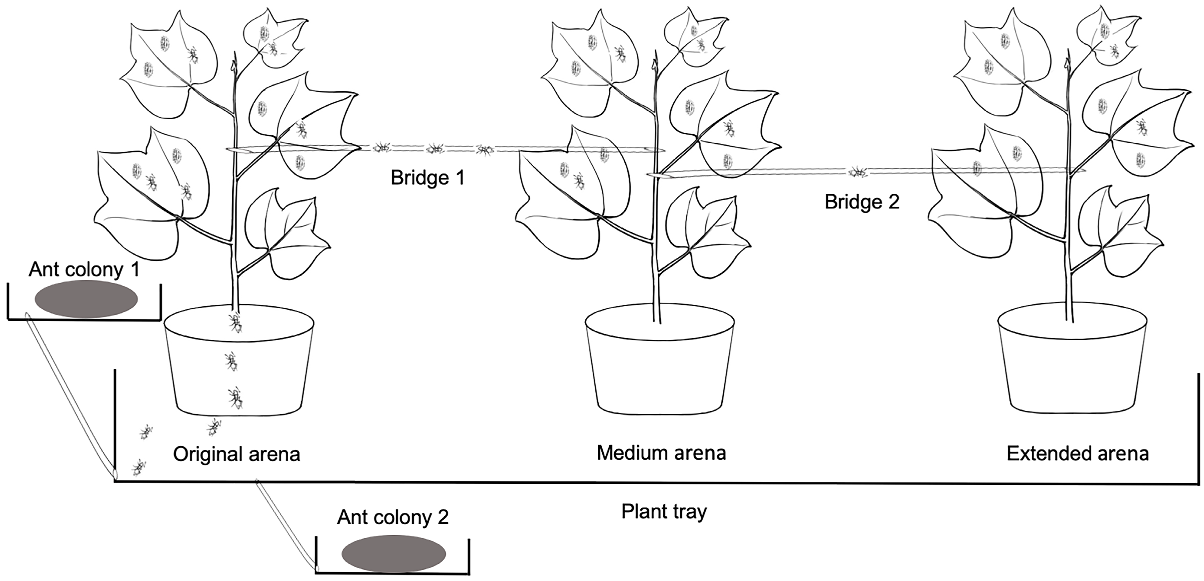

Figure S3 Graphical representation of apparatus used in experiment 2.7. We modified this apparatus according to the study of Grangier and Lester (2014). This apparatus was used to test the effects of ant interference competition on ant exploratory activity, which was described in experiment 2.7. Three cotton plants were placed in a plastic tray and each plant was 20 cm apart. Each two contiguous plants were connected by a wooden bridge. Each plant was considered as a different foraging arena for ant colonies: original arena, medium arena, and extended arena. Each foraging arena represents a different hierarchical level of exploratory activity. Exploratory activity of ant in original arena was defined as the number of workers moving up and down the plant trunk in 3 minutes. Exploratory activity of ant in medium arena and extended arena was defined as the number of workers moving toward and back the plant on bridge 1 and bridge 2 respectively in 3 minutes.

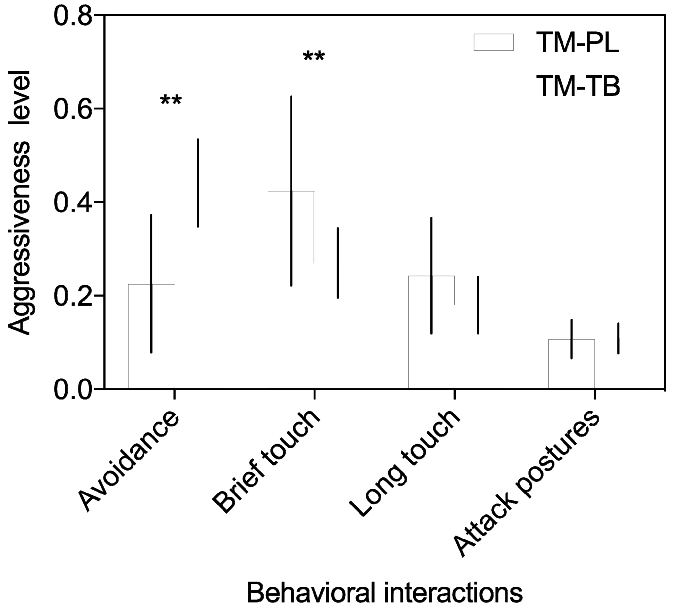

Figure S4 Effects of interference competition on ant aggressiveness level. The data are presented as the mean ± SD. Asterisk (**) on bars indicates significant differences between the treatments ($P < 0.05$ or $P < 0.01$).

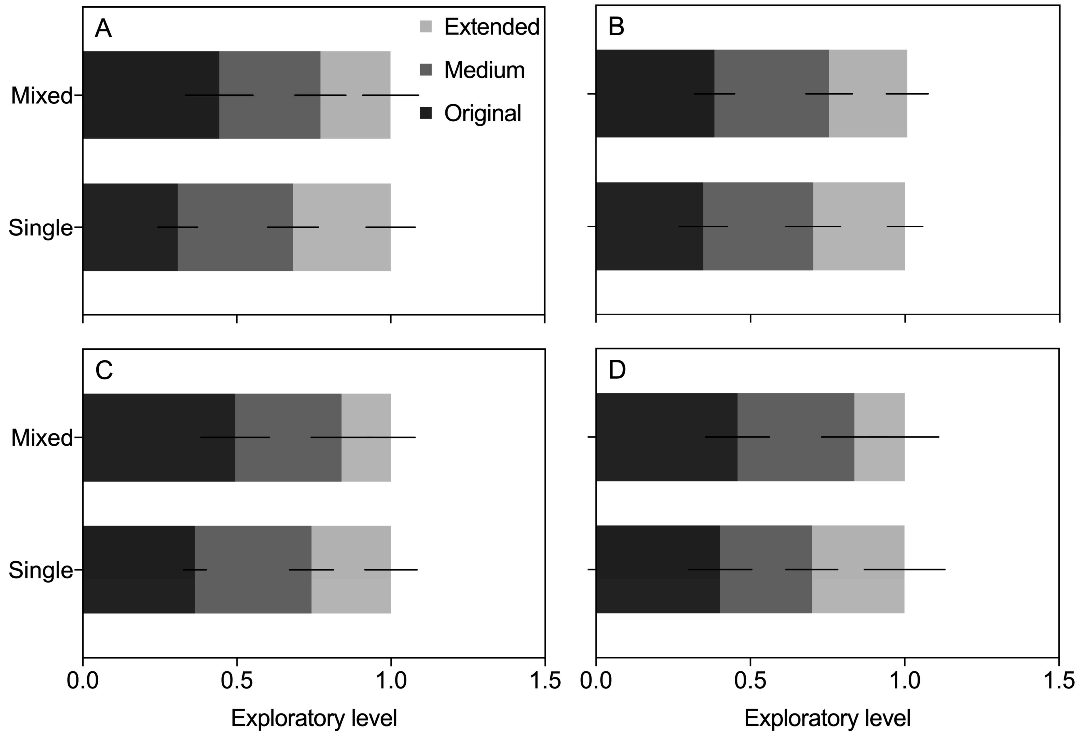

Figure S5 Effects of interference competition on ant exploratory level. A-C: *T. melanocephalum-P. longicornis* confrontation, B-D: *T. melanocephalum-T. bicarinatum* confrontation. A-B: Exploratory level of *T. melanocephalum*; C: Exploratory level of *P. longicornis*; D: Exploratory level of *T. bicarinatum.* The data are presented as the mean ± SD.
